# Effect of the aqueous extract of *Foeniculum vulgare* (fennel) on the kidney in experimental PCOS female rats

**Published:** 2014

**Authors:** Somayyeh Sadrefozalayi, Farah Farokhi

**Affiliations:** 1 *Department of Biology, Faculty of science, Urmia University, Urmia, I. R. Iran*

**Keywords:** *Estradiol-Valerate*, *Foeniculum vulgare*, *Kidney*, *Rat*

## Abstract

**Objective**: *Foeniculum vulgare* seed (*F. vulgare)* is an herbal plant which is used with phytoestrogene compounds for polycystic ovary syndrome (PCOS) treatment. In this research, renoprotective effect of the aqueous extract of *Foeniculum vulgare *(AEF) in experimental PCOS female rats is studied.

**Materials and Methods:** Forty female rats were randomly divided into five groups. The first group served as control, was injected with an equivalent volume (0.2 ml) of normal saline, and received normal diet. Animals in the second group were non poly cystic ovary syndrome (PCOS) rats which were treated with intragastric administration of aqueous extract of *F. vulgare *(150 mg/kg b.w.). In the third group, the rats were treated with intraperitoneal injection of estradiolvalerate (EV) (4 mg in 0.2 ml of sesame oil). The fourth groups were treated with EV and AEF (150mg/kg bw) with the same route. The fifth groups were treated with EV and AEF (100mg/kg bw). After 4 weeks of study, all of the rats were sacrificed, their kidneys tissues were processed for light microscopy, and some biochemical parameters of serum were measured.

**Results**: The mean values of blood urea nitrogen in PCOS rats treated with low dose of AEF and EV and non-treated, was significantly (p<0.05) increased compared with non-PCOS and PCOS rats treated with high dose of AEF. Moreover, histopathological changes of kidney samples were comparable in PCOS rats with respect to treated groups with AEF.

**Conclusion**: Aqueous extract of fennel seed showed the beneficial effect (especially at dose of 150 mg/kg b.w.) on renal function in PCOS rats.

## Introduction

Fennel (*Foeniculum vulgare*) is a well known Mediterranean aromatic plant, which is used in traditional medicine and as a spice. Diuretic, analgesic, and antipyretic activities have also been found in the fennel fruit as well as antioxidant activity (Choi et al., 2004 ▶). Essential oils are mainly concentrated in the mericarps and provide the unique aroma and taste (Siahi et al., 2009 ▶). Phytoestrogene is an active biological substance which can act similar to estrogen (Siahi et al., 2009 ▶). It also increases milk flaw and libido and relives menopausal symptoms in women (Khani et al., 2011 ▶). Both infusions and essential oils obtained from the fruits and the aerial parts of the plant, in fact, are included in the herbalist armamentarium for their relaxant, estrogenic, analgesic, and anti-inflammatory properties (Ostad et al., 2001 ▶). 

Estradiol valerate is a synthetic ester from 17β-estradiol which behaves as a prodrug, being cleared by esterase in blood plasma and the liver into 17β-estradiol and valeric acide (Düsterberg and Nishino, 1982 ▶). Polycystic ovary syndrome (PCOS) is a metabolic and reproduction disorder with characteristic features including hyperandrogenism, irregular menstrual, insulin resistance, obesity, hirsutism, and acne (Albert-Puelo ,1980 ▶). Anovulation due to PCOS is the most common cause of infertility in women (Engman et al., 2005 ▶). Long-term PCOS increases the risk of hyperplasia, endometrial cancer, metabolic syndrome, type 2 diabetes and cardiac arrest during sleep (Franks et al., 1995 ▶).

PCOS women are one of the groups in high-risk of type 2 diabetes (Mirzayi and Kazemi, 2007 ▶). Majority of diabetic patients are at risk of kidney disease (Castro et al., 2009 ▶; Chadban, 2010 ▶). Metabolic syndrome and hormonal disturbances are prevalent findings among PCOS women. Metabolic syndrome is a group of conditions including obesity, high blood pressure, blood fat abnormality, and borderline high blood sugar and insulin levels, which together increase chances of cardiovascular disease and type 2 diabetes (Lau et al., 2006 ▶; Mirzayi and Kazemi, 2007 ▶). Most of diabetic patients have kinds of kidney disorders (Castro et al., 2009 ▶). Administration of estradiol valerate causes further increases in lipid levels, which lead to progressive renal injury (Stevenson et al., 2000 ▶). Nowadays, many medicines are used for PCOS treatment (Robert et al., 2008 ▶) but the use of herbal medicines has always been considered. The aim of this research is the study of the renoprotective effect of the aqueous extract of *Foeniculum vulgare* in PCOS female rats.

## Materials and Methods

**Preparation of plant and extraction**

*Foeniculum vulgare* seeds were bought from market and identified by botanists in the Herbarium of Biology Department at Urmia University with herbarium number of 4843. The samples were ground by an electrical mill. The aqueous extract was prepared by cold maceration of 100 g of powdered in 400 ml of distilled water for 24 h. Then, the extract was filtered, concentrated, dried in vacuum, and the residue was stored in a refrigerator at 2-8 °C for use in subsequent experiments. 3.2 g dried *F. vulgare* powder was obtained per 100 g *F. vulgare* seeds. This powder was stored at 4 ºC until required for use. For the preparation of gavaged extract, this powder was solved in specific volume of normal saline. Multiple-doses of *F. vulgare* at doses of 150 mg/kg b.w. (body weight) and 100 mg/kg b.w. administered (European Food Safety Authority (EFSA), 2009). 

**Chemicals and drugs**

Estradiol valerate was purchased from Abureyhan Company. Normal saline and citrate buffer were acquired from Iran Darupakhsh Company, ethanol from Pakdis Iran Company, and other materials were purchased from Merk Company, Germany.

**Experimental protocol**

The current study was conducted on 40 healthy adult female Wistar rats weighing 200±20 g. These animals were purchased from Pasteur Institute, Iran and kept in animal house of Urmia University. They were kept at 20±5 ºC, relative humidity of 30±5% and light/dark cycle for 12 h. Animals were randomly allocated to five groups and treated as follows:

**Biochemical analysis**

At the end of the study, the animals were sacrificed and blood samples were transferred to tubes and immediately centrifuged (2500 rpm/10 min). Serum samples were stored at -20 ºC in form of frozen for biochemical analyses. Urea and creatinine were determined with the use of commercially available kits (Pars Azmoon, Tehran, Iran). 

**Histological study**

For histological study, the kidneys were removed and fixed in formol 10%. After tissue processing, the samples were blocked in paraffin. Transverse serial sections (5-μm thick) were prepared and 1/10 sections selected, transferred to slides and stained by hematoxylin and eosin (H&E). The sections were studied microscopically for the evaluation of histopathological changes (Hemati et al., 2012 ▶). 

**Statistical analysis**

All values were expressed as mean±SEM. The differences were compared using one-way analysis of variance (ANOVA) followed by Tukeys multiple comparison tests. p-value<0.05 was considered significant .

## Results

**Figure 1:**
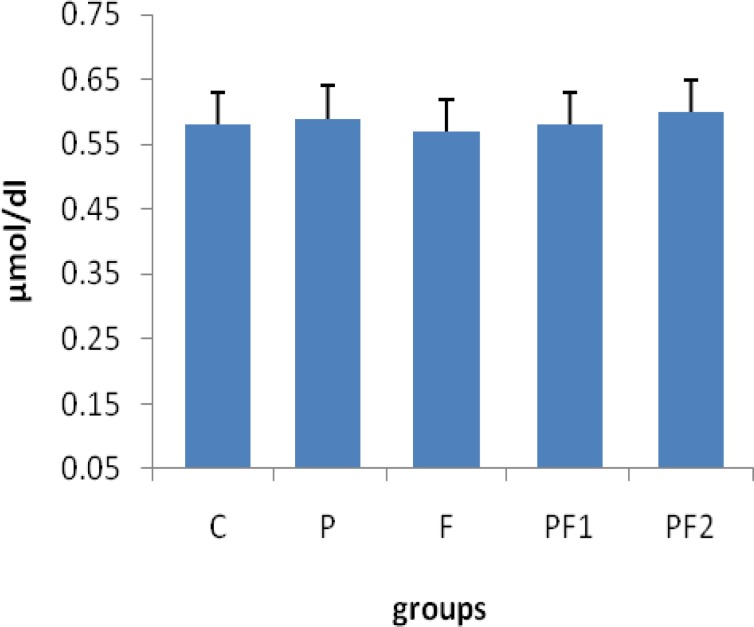
Serum creatinine levels in different groups. Data are expressed as mean±SEM. Each group consisted of 8 animals. C: non PCOS control rats, P: PCOS rats, F: non PCOS control rats received *F. vulgare* aqueous extract at 150 mg/kg b.w., PF1: PCOS rats were treated with *F. vulgare* at 150 mg/kg b.w., PF2: PCOS rats administrated with *F. vulgare* at 100 mg/kg b.w. There was no significant difference in serum creatinine

**Figure 2:**
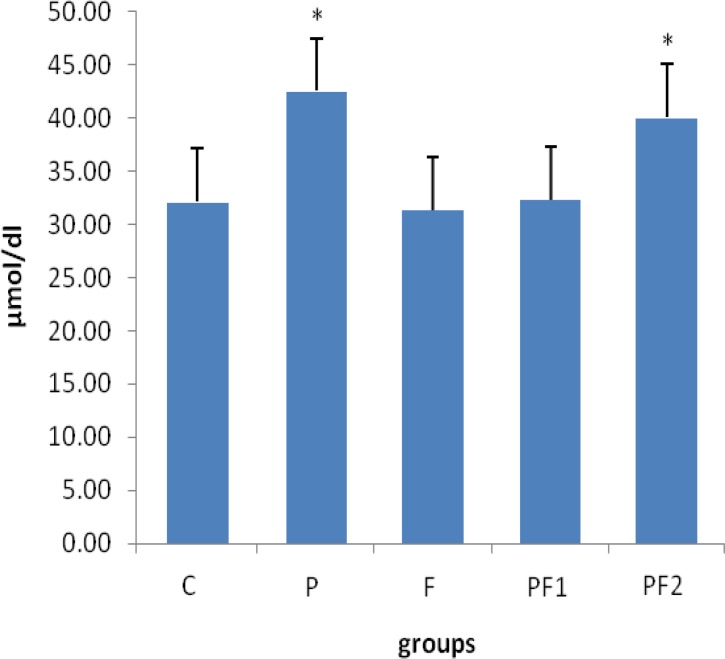
Serum urea levels in different groups. Data are expressed as mean ± SEM. Each group consisted of 8 animals. C: non PCOS control rats, P: PCOS rats, F: non PCOS control rats received *F. vulgare* aqueous extract at 150 mg/kg b.w., PF1: PCOS rats were treated with *F. vulgare* at 150 mg/kg b.w., PF2: PCOS rats administrated with *F. vulgare* at 100 mg/kg b.w. * indicates the significant difference between groups P and PF1 *vs.* control group (p<0.05).

Figure 1 summarizes the results of analyzing serum creatinine in different groups. This figure shows that serum creatinine levels did not change in different groups. While Figure 2 shows that level of urea in PCOS rats was significantly (p<0.05) elevated in comparison with control groups. Furthermore, serum urea levels were decreased in PCOS rats which were treated with *F. vulgare* at dose of 150 mg/b.w. Serum level of urea remained elevated in the PCOS rats which were treated with *F. vulgare* at dose of 100 mg/b.w. compared with control groups.

**Figure 3:**
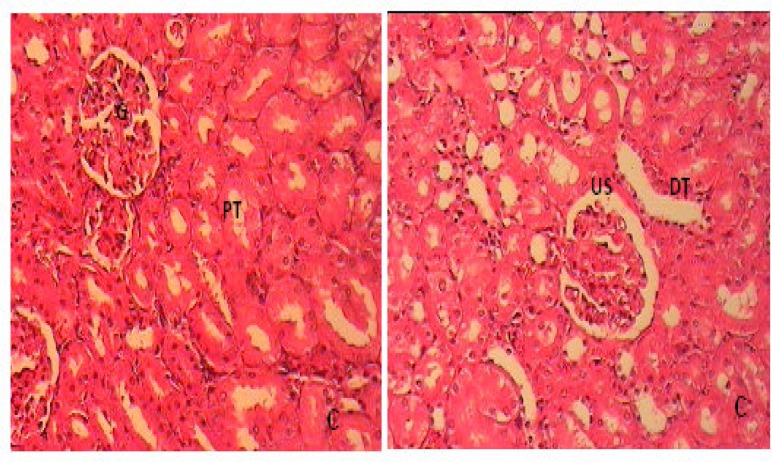
Cross section of kidney in C: control non PCOS rats (H&E×100), G: glomerulus, PT: Proximal convoluted tubules, DT: Distal convoluted tubule, US: Urinary space

Histopathology findings: Histology of kidney in control and non PCOS rats which was treated with extract of *F. vulgare *(150 mg/kg b.w.) showed normal structure (Figure 3).

**Figure 4:**
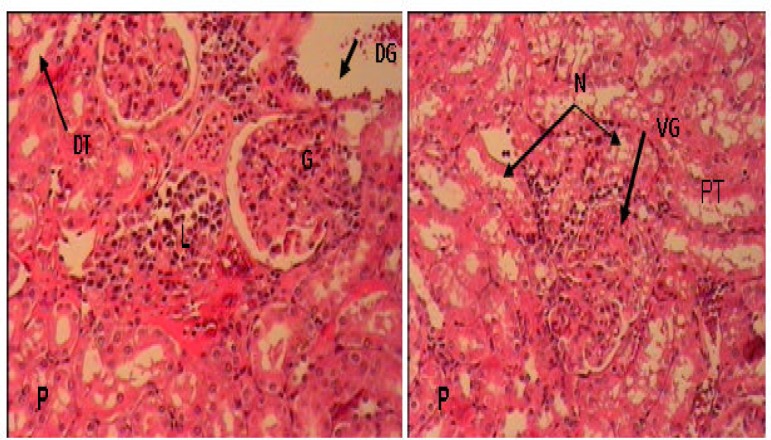
Cross section of kidney in (P), PCOS rats (H&E×100), G: glomerulus, PT: Proximal convoluted tubules, DT: Distal convoluted tubule, L: Lymphocyte, DG: Destroyed glomerulus, VG: Vacuolated glomerulus, N: necrosis in proximal tubule

**Figure 5:**
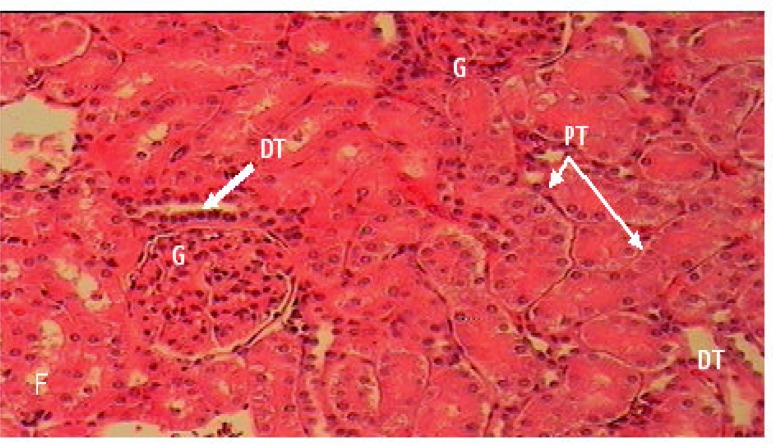
Cross section of kidney in (F) non PCOS rats treated with aqueous extract of* F. vulgare* at 150 mg/kg b.w. dose, (H&E×100) , G: glomerulus, PT: Proximal convoluted tubules, DT: Distal convoluted tubule

**Figure 6:**
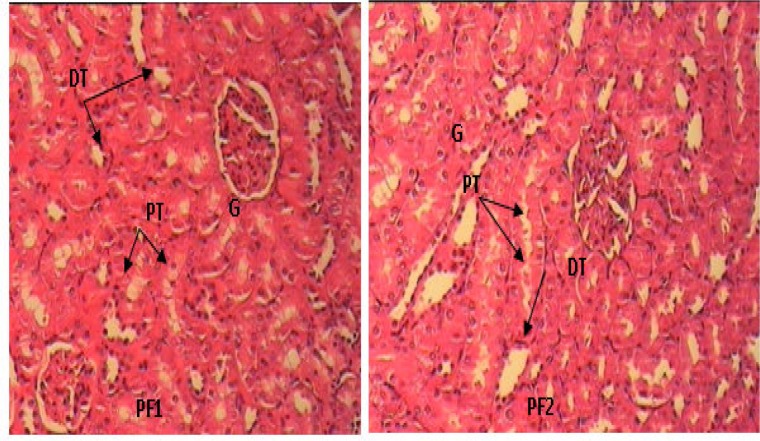
Cross section of kidney in PCOS rats treated with aqueous extract of* F. vulgare* at 150 mg/kg b.w. dose in PF1 and, at 100 mg/kg b.w. dose in PF2 rats (H&E×100), G: glomerulus, PT: Proximal convoluted tubules, DT: Distal convoluted tubule

In PCOS rats, kidney sections showed mild thickening of the basement membrane along with mild change in the density of Mesangial cells, atrophy of glomerular capillaries with increased Bowman's space (urinary space), and acute tubular necrosis (ATN). Moreover, presence of lymphatic cells in the connective tissue of around tubules indicates the presence of inflammation in the kidney (Figure 4). The groups that were treated with extract of *F. vulgare* (150 and 100 mg/kg b.w) showed features of healing, i.e., normal glomerulus, normal basement membrane, and capillaries (Figure 5). Moreover, Bowman's space (urinary space) and acute tubular necrosis (ATN) were improved towards normal condition after treatment with *F. vulgare* (150 and 100 mg/kg b.w.) (Figure 6).

## Discussion

In adult women with PCOS, metabolic syndrome is generally accompanied by hormonal disturbances such as elevated levels of serum androgen (Ehrmann, 2005 ▶). Moreover, they have a high incidence of hypertension and are at increased risk of renal diseases (Baylis, 1994 ▶). The kidneys can be affected by androgens. Mesangial cells, glomeruli, proximal tubules, and cortical collecting ducts contain androgen receptor (Quan et al., 2004 ▶). Testosterone can mediate renal injury in females. The concept that androgens may also be harmful to kidneys of females is important because postmenopausal women may produce more androgens as they age (Quinkler et al., 2003 ▶). 

Treatment of rats with dihydrotestosterone increases proximal sodium reabsorption and blood pressure (Baylis, 1994 ▶). Blood pressure can modulate renal injury. Thus, in PCOS models, elevated androgen levels, can mediate renal injury. Current study confirmed that renal injury in form of acute tubular necrosis with inflammations and presence of lymphatic cells in PCOS models. Also, in these groups, the mean level of urea was increased.

Estradiol has anti-inflammatory properties (Alvarez et al., 2002 ▶), which can protect glomeruli from increased transmission of the systemic blood pressure and protect kidneys against increased glomerular capillary pressure. Recently, it has been speculated that female sex hormones, such as estrogens, may be responsible for the lower susceptibility of women to progressive renal injury (Silbiger and Neugarten, 1995 ▶). External steroid hormones can be an effective treatment for repressing diabetic nephropathy (Antus et al., 2005 ▶). 

There is a growing interest, both in the industry and scientific research, for aromatic and medicinal plants because of their antimicrobial and antioxidant properties (Oktay et al., 2010 ▶). These properties are due to many active phytochemicals including flavonoids, terpenoids, carotenoids, coumarins, and curcumines (Ramawat and Goyal, 2008 ▶). These bioactive principles have also been confirmed using modern analytical techniques (Boskabady et al., 2002 ▶; Choi et al., 2004 ▶). Herbal drugs and essential oils of fennel have hepatoprotective effects (Ozbek et al., 2003 ▶). Phytoestrogens which have been used as estrogenic agents has been considered in treating of metabolic and hormonal abnormalities of women with polycystic ovary syndrome (PCOS) (Bhathena et al., 2002 ▶). Phythoestrogen replacement may have a protective effect against oxidative stress and renal disease. Phytoestrogens are structurally similar to endogenous estrogen and have affinity to estrogen receptors. 

There are various types of phytoestrogens such as isoflavones, prenylated flavonoids, and coumestans. Essential oils of the fruits of fennel (*Foeniculum vulgare*) constituents possess antimicrobial and antioxidant activities (Abdelaaty et al., 2011 ▶). Both infusions and essential oils obtained from the fruits and the aerial parts of the plant, in fact, are included in the herbalist armamentarium for their relaxant, estrogenic, analgesic, and anti-inflammatory properties (Boskabady et al., 2002 ▶). Flavonoid phytochemicals act as both agonists and antagonists of the human estrogen receptors which elicit antagonistic effects on estrogen signaling independent of direct receptor binding (Rosenblum and Saubern, 1993 ▶). Estradiol is usually thought to be renoprotective. 17β-estradiol inhibits apoptosis and transforming growth factor activity and expression (Negulescu et al., 2002 ▶). However, with low nitric oxide and high angiotensin II, estradiol exacerbates renal injury. In addition, in women, oral contraceptives increase blood pressure and albumin excretion and occasionally result in hypertension and renal damage (Ribstein et al., 1999 ▶). Thus, *F. vulgare with* phytoestrogen components could protect kidney disorders which are revealed in this research. In these groups, all of renal injury symptoms were improved. Diuretic property of *F. vulgare* has been announced in traditional medicine (Bekhradi, 2004 ▶). Our findings revealed that *F. vulgare* increased kidney activity and decreased urea rate. In this case, our results confirm traditional medicine evidences. 

*F. vulgare* has a protective effect on the kidney during progressive glomerulosclerosis in the female PCOS rats. Further studies are clearly needed to assess the potential applicability of these experimental findings in clinical setting.
